# “Treat people with human dignity”: the perspective of older adults on the quality of geriatric rehabilitation

**DOI:** 10.1007/s41999-024-01065-z

**Published:** 2024-09-26

**Authors:** Anne L. Lubbe, Julia Schellekens, Margriet C. Pol, Wim G. Groen, Bianca M. Buurman, Cees M. P. M. Hertogh, Marjon van Rijn

**Affiliations:** 1 Department of Medicine for Older People, Amsterdam UMC, Vrije Universiteit Amsterdam, de Boelelaan, 1117 Amsterdam, The Netherlands; 2 Aging and Later Life, Amsterdam Public Health, Amsterdam, The Netherlands; 3 Vivium Zorggroep Naarden, Naarden, The Netherlands; 4 Research Group Occupational Therapy: Participation and Environment, Faculty of Health, Center of Expertise Urban Vitality, Amsterdam University of Applied Sciences, Tafelbergweg 51, 1105 BD Amsterdam, The Netherlands; 5 Faculty of Health, Center of Expertise Urban Vitality, Amsterdam University of Applied Sciences, Tafelbergweg 51, 1105 BD Amsterdam, The Netherlands

**Keywords:** Geriatric rehabilitation, Older adult perspective, Quality, Interview study

## Abstract

**Purpose:**

The aim of this study was to gain insight into the perspectives of older adults on the quality of geriatric rehabilitation (GR) during the trajectory of GR from admission until six weeks after discharge.

**Methods:**

We conducted a longitudinal qualitative study. Participants were interviewed three times: at the start of rehabilitation, at discharge, and six weeks after discharge. The data were analysed using a thematic analysis.

**Results:**

In total, 50 interviews were conducted, with 18 participants being interviewed multiple times. The following themes emerged: 1. A bond of trust with health care professionals (HCPs), 2. Being prepared and informed at all stages of GR, 3. Participants emphasise physical and occupational therapy rather than other aspects of care as comprising GR 4. Changing needs regarding (the extent of) involvement in decision-making, 5. Contact with family and peers.

**Conclusion:**

For older adults, preparation for and good organisation of rehabilitation and social interaction with HCPs and other older adults were found to be important for the perceived quality of GR. Social interaction is influenced by how HCPs engage with older adults in all the phases of the rehabilitation process. Older adults have varying preferences about involvement in decision-making during GR. These perspectives should be acknowledged and acted upon in clinical practice to further improve the quality of care in GR.

**Supplementary Information:**

The online version contains supplementary material available at 10.1007/s41999-024-01065-z.

## Key summary points

**Aim:**

The aim of this study was to gain insight into the perspectives of older adults on the quality of geriatric rehabilitation (GR) during the trajectory of GR from admission until six weeks after discharge.

**Findings:**

The following themes emerged: 1. A bond of trust with health care professionals (HCPs), 2. Being prepared and informed at all stages of GR, 3. Participants emphasise physical and occupational therapy rather than other aspects of care as comprising GR, 4. Changing needs regarding (the extent of) involvement in decision-making, 5. Contact with family and peers.

**Message:**

For older adults, preparation for and good organisation of rehabilitation and social interaction with HCPs and other older adults were found to be important for the perceived quality of GR.

**Supplementary Information:**

The online version contains supplementary material available at 10.1007/s41999-024-01065-z.

## Introduction

In geriatric rehabilitation (GR) with its growing demand, rising costs, constrained resources, and variations in daily practice, maintaining a good quality of care (QoC) is challenging [1]. Although QoC is defined in many ways, the World Health Organisation (WHO) defines QoC as ‘the degree to which health services for individuals and populations increase the likelihood of desired health outcomes’ [2]. In the context of GR, QoC is often defined in terms of effectiveness, with a focus on goals, discharge home, and level of functioning. This is underscored by the definition of geriatric rehabilitation: “Diagnostic and therapeutic interventions aimed at restoring or enhancing functional capacity in older individuals with disabling impairments” [3]. The rehabilitation trajectory occurs over a specific duration and involves identifying individual problems and needs, setting rehabilitation goals, and implementing interventions through a multidisciplinary team [4].

The provision of GR by the multidisciplinary team is a complex process that can benefit from improved coordination, communication, and continuity of care among different healthcare professionals (HCPs) [5, 6]. HCPs are defined as all professionals that are involved in care or treatment in geriatric rehabilitation (e.g. healthcare assistants, nurses, physicians, and paramedics) [7]. The Literature suggests that various factors such as client centeredness, client satisfaction during rehabilitation and therapeutic climate contribute to the outcomes of GR, such as earlier discharge, lower mortality rates, shorter hospital stay, reduced cognitive and functional decline, and increased independence in activities of daily living [8–10]. Numerous quality indicators for GR exist, including factors related to structure, such as using an unambiguous triage model or discussing discharge criteria upon admission, and several focusing on the process, such as systematically analysed reports and treatment intensity categorised by diagnosis groups [11]. However, the perspective on QoC in GR of individuals undergoing it has been underrepresented [12, 13]. These experiences are typically captured at a single point of time in rehabilitation. However, rehabilitation is a longitudinal and dynamic process, which can result in evolving experiences and perspectives over time.

With this study we aimed to fill this void by better understanding the perspectives of older adults undergoing GR and how these may change over time.

## Methods

### Study design

We conducted a longitudinal qualitative study involving multiple interviews with older adults to explore their experiences and perspectives regarding the quality of GR. Given our research aim, a longitudinal qualitative study was deemed the most appropriate method. [14] It enabled us to examine older adults’ perspectives across different phases of the rehabilitation process (start of rehabilitation, just before discharge, and six weeks after discharge), thereby capturing potential changes over time [15].

### Participant sampling

#### Research population

The study population consisted of participants who were prospectively recruited from four geriatric rehabilitation centers, located in the northwest of the Netherlands. Three of these centers have multiple locations where GR is provided. These centers are affiliated with the University Network of Care Organizations for Older Adults (UNO) Amsterdam. We used purposive sampling to ensure the inclusion of a diverse group of participants with regard to sex, age, and diagnosis [16]. The treating physician decided on the eligibility of participants based on the following inclusion criteria: (1) being at the start of the rehabilitation (within 1.5 weeks), (2) proficiency in understanding and speaking the Dutch language, (3) able to participated in a conversation (4) signed informed consent, and (5) intention to return to an independent living situation after GR.

#### Participant recruitment

The HCPs responsible for participant recruitment were elderly care physicians and (physio) therapists employed at one of the participating GR centers. They informed potential participants about the study and its aims. If a person agreed to participate and provided informed consent, the researcher contacted the participant, to provide further details, and address potential questions. Subsequently, an appointment was made for an interview. Additionally, as an alternative means of recruitment, posters were displayed within the participating GR centers. Older adults could then contact the researcher directly after which eligibility was checked with their treating physician.

### Data collection

Data were collected from March 2022 to March 2023, by AL and JS. The participants were interviewed three times throughout their rehabilitation journey. The first interview occurred at the start of the rehabilitation (within 1.5 weeks after admission), the second interview was held just before discharge (within 3 days before discharge), and the third interview was conducted six weeks after discharge. Semi-structured interviews were conducted, allowing participants to explore topics in-depth and elaborate on their thoughts [17]. A comprehensive interview topic guide (Appendix A) was developed, that included predetermined topics derived from relevant literature and structured based on key concepts identified in a recent scoping review on the quality of GR from a patient perspective [18]. The first two interviews were conducted face-to-face at the various rehabilitation centers and the third interview was held at the participants’ homes. All sessions were audio-recorded and transcribed verbatim. Data on participants’ sex, age, admission diagnosis, and discharge time were also collected.

### Data analysis

Thematic analysis was conducted to analyze, identify, and interpret patterns within the interview data [19]. This approach enabled a thorough data description, as well as the identification of emerging key themes and patterns [17]. The analysis followed the six phases outlined by Braun and Clarke [19] (Appendix B). Interviews were transcribed verbatim and read by the interviewers, inductive open coding was applied to code the transcripts. Codes were clustered systematically into the potential themes. All themes were reviewed and redefined. All interviews were checked based on the final coding structure. Data were managed and organized using MAXQDA version 2022. Discrepancies during coding were resolved through discussion with the research team.

### Quality procedure

The Consolidated Criteria for Reporting Qualitative Research (COREQ) checklist was used (see Appendix C). This checklist assists in ensuring that the study procedures are described with sufficient detail to enhance reliability and transparency [20].

### Ethical considerations

The Medical Ethics Committee of The Amsterdam UMC has confirmed that the regulations under the The Medical Research involving Human Subjects Act do not apply to this study (2021.0790). Written informed consent was obtained before the first interview.

## Results

The study comprised eighteen participants, fifteen were interviewed three times, two were interviewed twice, and one was interviewed once. In total, 50 interviews were conducted. Table 1 provides an overview of the participants. The ages of the participants ranged from 63 to 94 years, with ten participants being female. The admission diagnoses that required GR were as follows: stroke (*n* = 5), orthopedic surgery (*n* = 5), COPD (*n* = 4), post-IC (*n* = 1), abdominal surgery (*n* = 1), and vascular disease (*n* = 2). The length of stay in the rehabilitation centers varied from 12 to 184 days. The participants were referred from different acute hospitals.

**Table 1:** 1 = first phase interview admission (± 1.5 week) GR, 2 = second phase interview discharge GR (± 3 days), 3 = third phase interview 6 weeks after discharge (± 1 week)

Code participant	Sex	Age (years)	Living situation	Diagnose	Rehabilitation centre	Length of stay (days)	Interviews completed
P1	F	74	Widow, living alone	Stroke	A	68	1, 2, 3
P2	M	80	Widower, living alone	Orthopedic surgery	B	12	1, 2, 3
P3	F	79	Widow, living alone	COPD	B	12	1, (lost to follow up)
P4	M	79	Widower, living alone	Stroke	B	18	1, 2, 3
P5	F	63	Living together with partner	Abdominal surgery & Multiple Scleroses	A	22	1, 2, 3
P6	F	81	Living alone	Orthopedic surgery	B	21	1, 2, 3
P7	M	63	Living together with partner	COPD	B	31	1, 2, 3
P8	M	85	Living together with partner	Orthopedic surgery	C	50	1, 2, 3
P9	F	71	Living together with partner	Post-IC	A	30	1, 2, 3
P10	F	94	Widow, living alone	Orthopedic surgery	C	33	1, 2, 3
P11	M	81	Widower, living alone	Stroke	C	42	1, 2, 3
P12	F	77	Widow, living alone	Orthopedic surgery	C	35	1, 2, 3
P13	F	89	Widow, living alone	Stroke	B	34	1, 2, (cognitive impairment after discharge)
P14	M	65	Living alone	Stroke	B	126	1, 2, 3
P15	M	82	Living together with partner	Vascular disease	D	184	1, 2, † (after discharge)
P16	M	78	Living together with partner	COPD	D	27	1, 2, 3
P17	F	70	Widow, living alone	COPD	D	35	1, 2, 3
P18	F	70	Living together with partner	Vascular disease, amputation	D	40	1, 2, 3

Based on data from 50 interviews, our analysis identified the following themes related to the quality of GR from the perspectives of older adults: 1. A bond of trust with HCPs, 2. Being prepared and informed at all stages of GR, 3. Participants emphasise physical and occupational therapy rather than other aspects of care as comprising GR, 4. Changing needs regarding (the extent of) involvement in decision-making, 5. Contact with family and peers.

### 1. A bond of trust with HCPs

Participants experience a state of dependency and mentioned that this dependency creates a greater need for a bond of trust.

Therefore, appropriate communication, both verbal and nonverbal is important. Engaging in eye level conversations, symbolises mutual respect and equality. However, participants begin their rehabilitation in a wheelchair, and participants mentioned the feeling of being looked down upon when being in a wheelchair, which feels disheartening and uncomfortable.

“Because I’m sitting low, the doctor squatted down to tell me, so that we were at the same level, which I found quite nice. And just everything in a very sympathetic manner.” (P1, t2).

Throughout the entire rehabilitation trajectory, participants consistently find it bothersome and belittling when they are not treated as equals. This is also reflected in language use, such as the use of diminutives.

“What I said is that I already feel inferior, and I think I feel this more intensely than perhaps others. So that difference is already significant and is then emphasized by saying ‘you just stay comfortably lying down' [using a patronizing voice].’’ (P6, t1).

During rehabilitation, participants develop a bond with the HCP involved in their care and treatment. The presence of familiar faces on the ward fosters a sense of connection and enhances the patient-HCP relationship. As the rehabilitation progresses, participants feel that they are truly known and understood by the HCP, which further strengthens the bond of trust between them.

Of course, I wanted to say goodbye to everyone on the ward. I had already said goodbye to some, because I knew they wouldn’t be there on Saturday, or maybe they knew it too and thought to come by for a visit. That was really nice. You do develop a kind of bond with those people after so many months. (P1, t3).

HCPs are crucial in motivating participants in GR. Participants expressed that the motivational aspect during the GR trajectory is particularly impactful and serves as an extra source of motivation for their recovery. According to the participants, the combination of an HCPs empathy and encouragement has a positive effect on the rehabilitation.

“They’re just open and honest. And, well, they are a bit, of course, a bit strict at times, giving you a kick in the backside now and then.” (P14, t3)

The relationship between HCPs and participants is strengthened for the participants when the professionals take the time to engage in meaningful interactions. This can take various forms, such as sharing a cup of coffee to discuss personal topics like vacations, or actively listening to the participant’s stories and concerns, taking the time to listen to what happened with the participants.

These small gestures of taking time for personal interactions reinforce the participant's sense of being seen as a human, beyond the medical condition(s), and contribute to a positive and supportive rehabilitation environment.

“Well, what I appreciated was that, um, I’m right in the middle of mourning, but I could tell them everything, and they weren't impatient at all, […] they just listened to you. I found that very comforting.”(P12, t1).

Despite the high workload, participants place great importance on the fulfillment of tasks, for example, support in taking a shower, because it allows them to progress in their rehabilitation and maintain a sense of continuity. They rely on HCPs to follow through on their commitments and expectations. When tasks are completed as promised, it reinforces the participants' trust and confidence in the HCP’s ability to provide effective care. As widely acknowledged, the staff shortage is also experienced by the participants, leading to longer waiting times, which, unfortunately, affects the QoC from the perspective of participants.

“Sometimes there's one staff member, sometimes two, and on Saturdays, there are definitely two or three. But then they leave at 3:30 PM, and only one remains. For three units, that’s just not feasible. If you mention it, they tell you to wait a bit, but sometimes you end up waiting for an hour.’’ (P16, t2).

### 2. Being prepared and informed at all stages of GR

The following subthemes, specific for the stages during GR, emerge from our longitudinal interview study: Being involved in preferred placement, Transfer from the hospital to the rehabilitation center, Prior knowledge and experience, First impression, Updates on progress, and A smooth transition home.

#### Being involved in preferred placement

Once the choice has been made to go to a rehabilitation center after hospital discharge, participants have a preferred place where they want to go. The place of preference is sometimes determined by previous rehabilitation experiences such as a visit to a rehabilitation center, stories from relatives, but also the location. Participants consider the location significant because they prefer to remain in their familiar surroundings or choose a facility close to their family, enabling them to receive visitors. Participants experience it as pleasant when they are involved in the choice of the location of rehabilitation. However, participants also are aware that places is scarce and are happy that a place has been found for them.

#### Transfer from the hospital to the rehabilitation center

After a rehabilitation location is found, the participants value the transfer from the hospital to the rehabilitation center. Participants considered the practical aspects of the transfer, such as transportation arrangements and logistical details, important considerations. Participants expressed surprise and some frustration when they had to manage these arrangements themselves. They valued guidance and support in navigating the transfer process.

“I was not allowed to take the ambulance because I was too good. I couldn’t even walk, but I was deemed too good. Then they said, ‘You’ll have to take a taxi, and you’ll have to pay for it upfront.’ […] I'm not going to pay for that. Well, um, then I went by car with my husband. Well, I had to recover for two days from that, it was so heavy.” (P9, t1).

#### Prior knowledge and experience

Participants had prior knowledge or experiences with rehabilitation through personal experiences or stories from others. Prior knowledge shaped their expectations of the rehabilitation process. However, most participants experienced GR for the first time. Participants expressed the importance of receiving information about the rehabilitation trajectory while still in the acute setting or the first days in the rehabilitation center. Having access to this information allowed participants to have a better understanding of what to expect during rehabilitation.

#### First impression

According to the participants, the first impression and conversation are crucial. If participants experience an unfortunate start, it was referred back to during the second and third interviews. However, a negative first impression is not always reversed into a positive experience. Participants stated that having a positive and welcoming atmosphere upon entrance is essential in establishing trust and confidence. The timing of these interactions also influences their lasting impact on individuals.

“Well, then I had an intake interview, which I was actually way too tired for, but I still had to answer those questions.’’ (P9, t1)

When participants arrive at the rehabilitation center, they have numerous conversations with different disciplines of HCPs. Which can become overwhelming, making it challenging to listen and absorb all the information at once. Setting boundaries for participants is then experienced as difficult, because as a participant in GR one is not at home and it feels like they have to follow the rules and schedule of the HCPs as a participant.

“I had an intake conversation with the doctor and another woman. They explained to me how things worked here, basically. You have to absorb everything, including expectations. It’s not immediately like, “Let’s dive right into it.” It’s also about them getting used to me and me getting used to them. So, that was all favorable. But in the afternoon, they didn’t really do anything. I just lay here and felt quite exhausted, so I closed my eyes for a bit. And otherwise, I just waited to see how the whole process unfolded.’’ (P15, t1).

#### Updates on progress

As the rehabilitation progresses, participants often feel uninformed about the specific rehabilitation goals that have been set for or with them. They experience a lack of communication and updates regarding these goals, and are unsure about what they are working towards and how their progress is being measured.

“But they don’t tell me along the way what they are going *to do* from the beginning, so I have no idea what they're working on.” (P5, t2)

#### A smooth transition home

A key recommendation highlighted by participants is the importance of a smooth transition to home and an ongoing involvement of HCPs who provide post-rehabilitation care. During the transition back home, participants greatly appreciated the offer of a home visit from a HCP. This gesture was perceived as supportive and provided reassurance and ongoing guidance as they resumed their daily lives outside of the rehabilitation center.

“The occupational therapist had visited *here* a few weeks earlier and provided some additional tips. That was helpful; I found it beneficial.’’ (P1, t3)

### 3. Participants emphasise physical and occupational therapy rather than other aspects of care as comprising GR

Therapy (physiotherapy and occupational therapy) was considered a crucial component of the participants’ rehabilitation and therefore participants expect to be adequately informed about this process, for example having a structured schedule for therapy sessions was emphasised, and participants found it beneficial to have the schedule documented on paper.

“Yes, there’s a nicely printed document of that; it outlines exactly how *to do* it, and it just works fantastically.” (P7, t2)

This provided clarity and helped them prepare and engage effectively in therapy. Additionally, participants stressed the importance of organisation, ensuring that therapy sessions were easily accessible without unnecessary obstacles or delays.

Participants in our study do not doubt HCPs' expertise because that’s what HCPs are trained for. Regarding the quality of therapy, participants provide limited feedback, mentioning only the variety of exercises and environment as positive.

“Yes, I find it difficult to judge whether I found it of good quality. I got the impression that it was, but I am not educated enough to assess that.” (P4, t3)

Some participants do not perceive care as part of their rehabilitation. According to participants rehabilitation is provided by physiotherapists and occupational therapists, while care is provided by nurses; in the eyes of participants, these are separate aspects. Being adequately prepared and informed about what to expect during the GR process helped them approach the rehabilitation with a more open mindset and willingness to actively engage in the process.

“But the physiotherapist ensures that I get back on my feet well. So that's the most important thing… The nurses are also doing their best, but well, they don’t make sure I get back on my feet.” (P7, t1).

### 4. Changing needs regarding (the extent of) involvement in decision-making

Upon their arrival at the rehabilitation center, participants express appreciation for having many things arranged for them. However, as the rehabilitation process unfolds, participants desire to have more control and autonomy.

“Yes, it’s still being arranged, but I’m okay with that. Maybe when I’m further along, I might want a bit more. That could happen. But at the moment, I’m satisfied with it.” (1A, T1).

“Well, it’s quite a struggle to have control in your own hands. We talked about my aversion to being patronized last time as well. There have been times when that was the case, so to speak.” (P6, t2).

During the first days, when goal-setting conversations occurred, participants expressed that these goals do not always resonate with their wishes. It appears that setting goals can be challenging for participants, and in some cases, the HCP may take over this task. On the other hand, participants have clear and specific goals in mind that they actively want to work on during their rehabilitation trajectory.

“For me, for the quality of rehabilitation care, they must encourage me to pick up my routines again as soon as possible, to regain independence.” (P10, t1)

The experienced quality of the rehabilitation is influenced by how HCPs respond to participants' characteristics. Acknowledging and supporting participants' individual coping mechanisms during rehabilitation is crucial. Participants stated that listening to their preferences and involving them in the decision-making processes is essential. However, some participants have a passive approach, allowing things to happen to them, while others may have clear expectations and a desire to actively participate in their rehabilitation process.

“That is also very important, the individual’s desire that um, how the care is adjusted, even a little bit, in consultation with the person it concerns. That is also very important because people can have a certain idea, like, ‘I want it this way’.” (P1, t1).

Throughout the rehabilitation process, efforts are made towards discharge, and participants prefer to have a say in the discharge timing and also receive the necessary information about it.

“Especially when people are leaving, it's important to indicate how the rest of the process will unfold and for them to be informed about home care and which organization from home care will be involved. It would be beneficial to have a more extended notice, even if it's just an estimate, about when you might be able to go home so that you don't get informed two days in advance that you’re allowed to go home tomorrow or the day after.” (P17C, t3).

### 5. Contact with family and peers

Participants who are open to contact with fellow older adults experience this as very pleasant. On the other hand participants indicate that they do not want to seek contact with others, but they attribute that to their attitude. If contact is desired, the participants also indicate that there must be an opportunity for this, for example, a room where people can sit.

“Well, a pleasant and spacious environment, that’s very important to me. It encourages patients to sit together more often.” (P3, t1)

The opportunity to meet people, such as eating together, is also perceived as positive. Meeting and seeing peers also provides new insights. It is a way of acquiring new knowledge and reflecting on yourself. This can contribute to the rehabilitation process.

“There was a young man, about 55 years old, who had had his leg amputated, and he was kind of a fellow sufferer. I used to talk to him before his leg was amputated. I talked a lot with that man, and he kept telling me, “Just do it because you’ll be relieved of the pain, and that’s already a big plus. It’s just unbearable like this.” (P15, t2).

Finally, for participants in our study, there is also a social aspect where friendships develop during the rehabilitation process, which can make the rehabilitation a lot more pleasurable.

After discharge, participants also miss a certain level of social contact.

“That is quite confronting because suddenly you are alone for a large part of the time.” (P1, t3)

There were no restricted visiting times in the rehabilitation centers where the interviews took place, visitors were always welcome, which is experienced as positive, especially if the participants compare it with the hospitals.

“With visiting hours, it’s quite flexible, as long as there’s no therapy. Then anyone can come.” (P1, t1)

Participants emphasised the importance of having designated spaces for receiving visitors during their rehabilitation. Specifically, participants expressed appreciation for the option of having visitors join them in the restaurant. By having designated areas for receiving visitors, participants felt that their social connections were nurtured and maintained during their rehabilitation.

## Discussion

In this study, we explored the longitudinal perspectives of 18 older adults regarding the quality of geriatric rehabilitation. We found the following themes emerging from the data: 1. A bond of trust with HCPs, 2. Being prepared and informed at all stages of GR, 3. Participants emphasise physical and occupational therapy rather than other aspects of care as comprising GR, 4. Changing needs regarding (the extent of) involvement in decision-making, 5. Contact with family and peers.

When we compare our findings to previous literature on quality of GR we find notable differences between the organizational and patient perspectives. Literature on quality of GR [3, 4, 11], to date mainly focusses on effectiveness, length of stay, and level of functioning. Successful rehabilitation is described by Holstege et al. [6] in terms of independence in activities of daily living at discharge, discharge to home, and length of stay. The metrics for quality of GR and successful rehabilitation from an organizational or professional perspective seem to focus on the efficiency of the patient flow within the GR departments. The themes that emerge from the current study on quality of care from the patient perspective indicate that older adults do not assess the quality and success of rehabilitation on metrics such as effectiveness, length of stay, and level of functioning. Although participants -of course- also have a goal of going home, empathetic human interaction and communication, by the HCP are crucial.

One of our main findings is that, the quality of GR primarily seems to reside in the way HCPs interact with older adults and inform them during the rehabilitation process. Participants explicitly mentioned the importance of forming a bond of trust and expressed changing needs over time for involvement in decision-making, underscoring the importance of applying a longitudinal design. On the other hand, the longitudinal approach confirmed the continued importance of being properly informed and prepared at key transition points in the GR trajectory. Successful or good quality rehabilitation from the older adults’ perspective seems thus to be comprised of static and dynamic factors centering around human interaction and communication.

Rehabilitation goals during inpatient rehabilitation are to regain functioning and independence in self-care and to return home. Rehabilitation goals appear to change over time. In previous literature, HCPs indicated that goals have to be related to discharge criteria [21]. Some individuals with physical limitations may have limited capacity to make informed treatment decisions and participate in decision-making, particularly during the early stages of rehabilitation [22, 23]. As rehabilitation progresses, and individuals become more independent, their wishes and goals take priority, and more value is placed on being involved in decision-making [24]. An invitation for decision-making is crucial for patients to establish effective communication [25]. Tijsen et al. [26] emphasised the importance of communication in building a therapeutic relationship. However, participants in our study noted that it is challenging to establish a good relationship with permanent staff members for a person- centered approach.

Janssen et al. [10] discuss the therapeutic climate that HCPs aim to promote and the information they provide. However, there appears to be a gap between what older adults actually understand and what is intended to be communicated; HCPs offer information and recognise its significance, yet it must be effectively communicated to older adults. Participants of our study emphasise physical and occupational therapy rather than other aspects of care as comprising GR, especially in the first round of interviews. Reasons for this are unclear, but might be related to the use of the term “rehabilitation” and the association of performing exercises and other activities supervised by HCPs. To assess the quality of care provided by the entire rehabilitation team, including nurses [7], subsequent interviews addressed each discipline within the treatment team separately, that was actually involved in the older adult's care.

Vaalburg et al. [27] studied the role of nursing in a goal-centered care setting. Assigning a unique role to nursing as a bridge between the older adult and the multidisciplinary rehabilitation team, the older adults gained more insight into the process and became more in control of the treatment goals of the multidisciplinary team.

Participants express a need for information about the rehabilitation process. For effective commencement and participation in rehabilitation, the provision of information and opportunities for dialogue are essential. Our recent scoping review about the perceived quality of GR, mainly comprised of studies with stroke patients [18], showed that older adults who participated in rehabilitation strongly desire to share their stories. Additionally, our review underscores the findings of our current study of the importance of providing necessary information to individuals and supporting rehabilitation that continues in their home setting. Information provision and space for dialogue contribute to the continuity of care. GR is a dynamic process that continuously changes but should offer stability, so called personal continuity. Personal continuity is an established fundamental principle of primary care. [28–30] An admission to GR in fact serves as an intermediary phase in primary care, and it would be beneficial if older adults perceive continuity throughout this entire process. The need for information about the GR and familiar faces in GR is relevant for the continuity of care. This is important throughout the entire process, from the trauma to home, and ideally, the system, with the barriers should change for the older adult, eliminating the barriers in the healthcare process.

### Strengths and limitations

This study has several strengths. First, the researchers developed a trustworthy relationship with the older adults through multiple interviews and visits to the rehabilitation centers. This allowed the researchers to discuss more delicate themes with the participants, in which the participants experienced the feeling of being heard. Second, the study’s longitudinal design allowed for the investigation of (the evolution of) perceived QoC during the GR trajectory. By conducting this study longitudinally, individuals remained actively involved in the studied situation, minimizing the potential for recall bias and enabling a more accurate understanding of their experiences. Third, the interviews were conducted in various GR healthcare settings. Furthermore, all interviews were coded independently by JS and AL. Investigator triangulation was used to reduce observer bias and improve the inter-judge reliability by adding breadth to the phenomenon of interest [31].

However, there are also some limitations to consider: The participants in this study were reliant on the care they received. Therefore, the possibility of social desirability in answering questions and a selection bias (participants were selected by their treating physician) should be taken into account. Finally, generalisability may be limited because we included only four care organisations in the Northwest of the Netherlands and precluded participation of patients with speech language or cognitive problems. However, we tried to include a diverse population regarding diagnoses, gender and age. In future research the local and culture differences should be included.

### Implications for practice

Our results have several important implications for current practice. Participants express that they define rehabilitation by means of receiving therapy, whereas HCPs do not want to make this distinction in the rehabilitation setting where everything (and everyone) is considered to be part of the rehabilitation process. The contributing role of non-therapists (e.g. nurses) to the rehabilitation process should be made more clear [32].

Second, the participants do not address the quality of HCPs in their evaluation of the QoC. They do not discuss the treatment itself (e.g. what type of exercises, with what intensity etc.) but rather assume the expertise of the HCP on this matter, for the treatment itself other indicators are necessary to measure the quality. Third, for the participants, QoC is more about the manner of interaction rather than solely relying on knowledge. These findings highlight a need for more focus on meeting the individual needs of the older adults during rehabilitation. Educational materials for HCPs may improve awareness of needs and perspectives of older adults in GR.

## Conclusion

Older adults appear to have a unique perspective on perceived quality of GR resulting in elements often lacking in traditional indicators or tools about quality of GR.

Discovering that adequate preparation and organisation of therapy, as well as meaningful social interaction, were deemed crucial for enhancing the perceived quality of GR according to the needs of older adults. Furthermore, participants experienced a strong desire for connecting with HCPs, and they feel that communication during the GR process often revolves around them rather than with them.

These perspectives should be acknowledged and acted upon in clinical practice to further improve quality of GR care.

## Supplementary Information

Below is the link to the electronic supplementary material.

Supplementary file1 (DOCX 30 KB)

Supplementary file2 (DOCX 33 KB)

## Data Availability

The data will be made available, from the corresponding author on reasonable request.
