# Supplementary material for: “Treat people with human dignity”: the perspective of older adults on the quality of geriatric rehabilitation
Source: Eur Geriatr Med. 2024 Sep 26;15(6):1783–92. doi: 10.1007/s41999-024-01065-z (PMC11631986; doi:10.1007/s41999-024-01065-z)
Supplement: Supplementary file 1 — Supplementary file1 (DOCX 30 KB) [file 41999_2024_1065_MOESM1_ESM.docx]

Appendix A

## Phase 1 interview

### Start

- Introductions patient and interviewer
- Discussing confidentiality
- Explain the purpose of the research is to capture clients' experiences regarding the quality of the provided geriatric rehabilitation care.
- The duration of the interview is approximately half an hour to an hour.
- Asking for permission for audio recording, mentioning confidentiality – start recording as soon as possible.
- Allowing the client to ask any questions beforehand.
- Recording age, gender, condition, location, and duration of stay.
- Can you briefly explain what happened?
- Why did the patient enter the rehabilitation program?
- Hospital admission date and reason
- Experience with previous rehabilitation programs

### Expectations

- Prior obtained information about the rehabilitation:
  - Was it clear?
  - Did you find the way it was received/communicated pleasant?
- Decision-making:
  - Were you allowed to participate in the decision-making regarding your treatment trajectory?
  - Were your ideas taken into consideration?
- What are your expectations for your rehabilitation program? (patient's expectation management)
  - What do you expect from the treatment?
  - What role will you play in this process?

### Perception on the process

- How do you experience the progress of your rehabilitation?
- Does the progress of rehabilitation align with your expectations?
- Is the rehabilitation plan transparent to you?
- Is the rehabilitation plan adaptable? Do you have a say in achieving your goals?
- Autonomy – Do you feel like you have control over the process?
- Do you receive enough support in physical, psychological, and social aspects? From whom? Treatment team – specific individuals from the treatment team to be asked (psychologist, occupational therapist, geriatrician, physiotherapist)
- What are the positive aspects?
- What could be improved?
- How are the environmental factors?
  - Family
  - Room/space facilities
  - Group sessions/treatments
- Adjustments based on habits, needs, and cultural preference?
- Do you feel that your personal needs are taken into account adequately?
- Feeling of privacy
- Did you feel comfortable during the rehabilitation process?
  - Home situation
  - Social network
- Expectations after discharge – who or what do you anticipate needing?
- How do you experience the treatment team?
  - Number of people
  - Communication (between patient and team)
  - Accessibility/availability
  - Collaboration (team functioning)
- Do you have a need for a patient panel?
- Communication between informal caregiver, patient and healthcare professional

### Outcome

- Are you satisfied with the entire care process? To what extent are you satisfied with the entire care process?
- Do you have any recommendations for care? What would you recommend
- Perception towards going home
- Are you confident about going home? If the answer is no: what do you need to gain more confidence in this?

### Quality

- What does your ideal rehabilitation process look like?
- What do you understand by quality of rehabilitation care?
- What are the requirements for this?
- Would you recommend this rehabilitation to family or friends?
  - If no: ask further what would need to be changed so that you would do this.

### End

- Would you like to say anything more about the care received?
- Brief summary of what has been discussed
- Thank you for cooperating

## Phase 2 interview

- Again ask verbally for permission to record the interview
- Any short explanation of the research again

### Introduction

- How have the past few weeks been here for you? / How are you?
- You indicated last time that these were your expectations/goals (see previous interview) how do you view this now?
- Match the course of your rehabilitation with your expectations?
- Have you run into any problems in the past few weeks during the rehabilitation process?
- How was the provision of information during rehabilitation?

### Treatment plan

- How did you experience the rehabilitation plan/treatment plan/rehabilitation schedule?
- Do you feel that you were able to participate in the treatment plan?

### Autonomy

- Did you feel you were in control during rehabilitation/How did you experience your participation in the rehabilitation process?
- In your opinion, did you have sufficient say in the rehabilitation process here?

### Treatment team

- What did you think of the treatment team?/How did you experience the treatment team?
  - How was the contact with your doctor
  - How was the contact with the physiotherapy? What did you think of the physiotherapy? What did you think of the frequency of physiotherapy?
  - What is the contact with occupational therapy
  - How was the contact with the nursing staff
  - Possibly a social worker? Was there enough space to tell your story/were you listened to by the staff
- Do you feel that the staff have enough time for you?
- Number of people – What do you think of the diverse treatment team? Similar faces or not?
- Communication (between patient and team) – How do you feel the treatment team communicates
- Are things discussed with you in an understandable way?
- Availability/accessibility – Is the treatment team easily accessible if necessary/Does the treatment team have enough time for you?
- Collaboration (functioning of the team) – How do the different healthcare workers work together?
- You have seen many different healthcare professionals, what does the ideal healthcare professional look like to you?

### Environment

- -When we look at the environment here, how do you feel?
- How was the possibility to receive visitors?
- What did you think of your room/room composition?
- What did you think of the group therapies (if any)
- Did you feel that your personal needs/wishes were taken into account?
- If you have a need for something, to what extent are your wishes met?
- How did you fill in your day here besides the fixed times?
- Privacy: What did you think of the privacy during your rehabilitation?
- Did you feel comfortable here?

### Home-situation

- How did you like the information provided about going home (if not satisfied, how would you have liked it differently)
- How do you feel about going home / Do you feel confident about going home?
- Do you need any adjustments in your home before you return home?
- If you look at the completion of the rehabilitation, are there still goals for home that you want to achieve, what do you need for that?

### Outcome

- Are you satisfied with the entire care process? To what extent are you satisfied with the entire care process?
- Have your treatment goals been achieved? What has contributed most to this
- Do you have any recommendations for rehabilitation?

### Quality

- Could you briefly describe your rehabilitation process that you have gone through (so far)?
- Are there any points for attention/improvement for you?
- What does your ideal rehabilitation process look like?
- If you could change something about your rehabilitation process what would it be?
- How do you look back on your rehabilitation process here?
- What do you understand by quality of rehabilitation care?
- Were there factors that hindered the quality of the rehabilitation for you?
- What are the requirements/which elements are important for this?
- Would you recommend this rehabilitation to family or friends? Why/why not?

### End

- Do you have any questions for me or something you want to share?
- Short summary of what has been discussed?
- Thank you for cooperating

## Phase 3 interview

### Outcome

- You have been home for a number of weeks now, how have the weeks been for you after the rehabilitation?
- (Let's go chronologically over the past period) We saw you just before departure, but how did the transfer go home?
- How did you experience the first days after returning home?
- Did you feel that during rehabilitation you were sufficiently prepared for your return home and further rehabilitation?
- To what extent did you receive information about further rehabilitation at home? (and from whom?)
- What did you think of the information you received regarding further home rehabilitation?
- What did you think of the way you were helped/accompanied during the discharge?
- Have you run into any problems in the past few weeks?
- You indicated at the beginning of the rehabilitation that your goal was …, do you think you have achieved that goal?
- Do you currently receive care at home? If yes which one
- Did you still have contact with (name of specific rehabilitation center) after discharge? If not, would you have liked it?
- From whom do you currently receive the most support?
- If you now look back on the entire rehabilitation process, what recommendations would you have for rehabilitation care?
- When you look back at the past period and the rehabilitation, what do you think of that?
- To what extent are you satisfied with the result achieved?
- Do you experience major differences between before and after the rehabilitation process?
- How did the rehabilitation process affect you?
- Were there aspects during your rehabilitation that you would have liked to have seen differently?
- Would you recommend the rehabilitation in (rehabilitation centre) to friends or family, why? What do you say about the rehabilitation in (mention rehabilitation centre), why someone should or should not go here?
- If I had to ask you one last time, now that you have received geriatric rehabilitation care yourself, how would you describe the quality of geriatric rehabilitation care?
- How do you see the future?
