# Supplementary material for: “Treat people with human dignity”: the perspective of older adults on the quality of geriatric rehabilitation
Source: Eur Geriatr Med. 2024 Sep 26;15(6):1783–92. doi: 10.1007/s41999-024-01065-z (PMC11631986; doi:10.1007/s41999-024-01065-z)
Supplement: Supplementary file 2 — Supplementary file2 (DOCX 33 KB) [file 41999_2024_1065_MOESM2_ESM.docx]

Appendix B: Six phases of Braun and Clarke

| **Phase** | **Procedure for each phase** |
| --- | --- |
| Phase 1: familiarizing with data | Interviews were transcribed verbatim and read by the interviewer to become familiar with the information obtained. The qualitative analyzing tool MAXQDA was used to manually code all transcripts. |
| Phase 2: generating initial codes | Inductive open coding -without using a pre-existing code frame- was applied to code the transcripts and establish initial themes. |
| Phase 3: searching for themes | Codes were clustered systematically into the potential themes and patterns to identify relationships between categories. |
| Phase 4: reviewing themes | All themes were reviewed and redefined in relation to the coded extracts to determine if the identified themes contain enough data to support them. |
| Phase 5: verifying and redefining themes | After analysis of all the transcripts by the first researcher (AL), a second researcher (JS) systematically examined and verified all transcripts and codes. In some cases, it was necessary to define, refine or revise themes, based on the assessment of the second researcher (JS). |
| Phase 6: producing the report | All interviews were checked based on the final coding structure. The most relevant quotes were labelled and translated to English to support key findings. |

Appendix C: Consolidated Criteria for reporting Qualitative Research Checklist (COREQ)

| **No** | **Topic** | **Guide question/description** | **Answer** | **Page no.** |
| --- | --- | --- | --- | --- |
| Domain 1: research team and reflexivity | | | | |
| *Personal characteristics* | | | | |
| 1. | Interviewer/facilitator | Which author/s conducted the interview or focus group? | AL and JS conducted all interviews | 4 |
| 2. | Credentials | What were the researcher’s credentials? E.g. PhD, MD | MSc | 1 |
| 3. | Occupation | What was their occupation at the time of the study? | PhD student, master student | 1 |
| 4. | Gender | Was the researcher male or female? | Female | 1 |
| 5. | Experience and training | What experience or training did the researcher have? | The researchers followed several qualitative research courses and courses on interviewing techniques. JS also conducted a previous qualitative study in which general practitioners were included. | 1 |
| *Relationship with participants* | | | | |
| 6. | Relationship established | Was a relationship established prior to study commencement? | No | 3/4 |
| 7. | Participant knowledge of the interviewer | What did the participants know about the researcher? e.g. *personal goals, reasons for doing the research* | The reason for conducting this research; gaining insight into the patient perspective on quality of geriatric rehabilitation care. Furthermore, the participants knew the researchers’ affiliation | 3/4 |
| 8. | Interviewer characteristics | What characteristics were reported about the interviewer/facilitator? e.g. *Bias, assumptions, reasons and interests in the research topic* | No further characteristics were reported than researcher’s affiliation | 4 |
| Domain 2: study design | | | | |
| *Theoretical framework* | | | | |
| 9. | Methodological orientation and Theory | What methodological orientation was stated to underpin the study? e.g. *grounded theory, discourse analysis, ethnography, phenomenology, content analysis* | Thematic Analysis | 5 |
| *Participant selection* | | | | |
| 10. | Sampling | How were participants selected? e.g. *purposive, convenience, consecutive, snowball* | Purposive sampling | 3 |
| 11. | Method of approach | How were participants approached? e.g. *face-to-face, telephone, mail, email* | Indirectly. The elderly care physicians working approached the participants | 3/4 |
| 12. | Sample size | How many participants were in the study | 18 participants | 5 |
| 13. | Non-participation | How many people refused to participate or dropped out? Reasons | three   - 1 no response for second interview - 1 cognitive impaired third interview - 1 dead third interview | 5-8 |
| *Setting* | | | | |
| 14. | Setting of data collection | Where was the data collected? e.g. *home, clinic, workplace* | In the rehabilitation centres and home setting | 4 |
| 15. | Presence of non-participants | Was anyone else present besides the participants and researchers? | In 4 interviews the partner was present | - |
| 16. | Description of sample | What are the important characteristics of the sample? e.g. *demographic data, date* | Patients had to have an indication for geriatric rehabilitation and be at the start of their rehabilitation process.  Furthermore, they had to speak and understand the Dutch language. Patients who were cognitively unable to have a conversation with were excluded. | 3 |
| *Data collection* | | | | |
| 17. | Interview guide | Were questions, prompts, guides provided by the authors? Was it pilot tested? | A interview guide was developed by two authors (AL, JS). Using semi-structured interviews, in which the interviewee was able to express his or he own thoughts and feelings in a thorough way. The interview guide was discussed with several other researchers and pilot tested. | 4 |
| 18. | Repeat interviews | Were repeat interviews carried out? If yes, how many? | Yes, 50 interviews were conducted | 5 |
| 19. | Audio/visual recording | Did the research use audio or visual recording to collect the data? | Audio recordings were used. | 4 |
| 20. | Field notes | Were field notes made during and/or after the interview or focus group? | Yes, some notes were made during the interviews | 4 |
| 21. | Duration | What was the duration of the interviews or focus group? | 45-60 minutes | - |
| 22. | Data saturation | Was data saturation discussed? | We have tried to reach data saturation. Saturation was reached in all phases. | - |
| 23. | Transcripts returned | Were transcripts returned to participants for comment and/or correction? | Due to pragmatic reasons (older patients without email address) transcripts were not returned. A member check with the participants was not conducted according to the standard procedure, i.e. by providing a written summary, but the interviews were immediately verbally summarized and discussed with the patient. | - |
| Domain 3: analysis and findings | | | | |
| *Data analysis* | | | | |
| 24. | Number of data coders | How many data coders coded the data? | Both AL and JS have separately coded all transcripts. Thereafter all codes were thoroughly discussed with the research team during data analysis | - |
| 25. | Description of the coding tree | Did authors provide a description of the coding tree? | Yes | - |
| 26. | Derivation of themes | Were themes identified in advance or derived from the data? | Derived from the data | 8 |
| 27. | Software | What software, if applicable, was used to manage the data? | Transcripts were coded in MAXQDA | 5 |
| 28. | Participant checking | Participant checking  Did participants provide feedback on the findings? | Patients who were interviewed did not provide feedback on the findings | - |
| *Reporting* | | | | |
| 29. | Quotations presented | Were participant quotations presented to illustrate the themes / findings? Was each quotation identified? e.g. *participant number* | Yes. Each quote contains a participant number and interview phase (e.g. P.., I..) | 9-17 |
| 30. | Data and findings consistent | Was there consistency between the data presented and the findings? | Yes | - |
| 31. | Clarity of major themes | Were major themes clearly presented in the findings | Yes | 9-17 |
| 32. | Clarity of minor themes | Is there a description of diverse cases or discussion of minor themes? | Yes | - |
